# A von Hamos-type hard X-ray spectrometer at the PETRA III beamline P64

**DOI:** 10.1107/S1600577519013638

**Published:** 2020-01-01

**Authors:** Aleksandr Kalinko, Wolfgang A. Caliebe, Roland Schoch, Matthias Bauer

**Affiliations:** a Department Chemie and Center for Sustainable Systems Design (CSSD), Universität Paderborn, Warburger Straße 100, 33098 Paderborn, Germany; b Photon Science DESY, Notkestraße 85, 22607 Hamburg, Germany

**Keywords:** von Hamos, high-resolution emission spectroscopy, HERFD-XANES

## Abstract

The design and performance of the high-resolution wavelength-dispersive multi-crystal von Hamos-type spectrometer at PETRA III beamline P64 are described. Extended analyzer crystal collection available at the beamline allows coverage of a broad energy range from 5 keV to 20 keV with an energy resolution of 0.35–1 eV. Particular attention was paid to enabling two-color measurements by a combination of two types of analyzer crystals and two two-dimensional detectors. The performance of the spectrometer is demonstrated by elastic-line and emission-line measurements on various compounds.

## Abstract 2

The design and performance of the high-resolution wavelength-dispersive multi-crystal von Hamos type spectrometer at PETRA III beamline P64 are described.

## 1. Introduction   

High-energy-resolution non-resonant hard X-ray emission spectroscopy (XES) is increasingly recognized as a powerful tool to determine the electronic and even geometric structure of matter by means of core-to-core (CtC) and valence-to-core (VtC) XES. Both methods have found many applications in chemistry (Zimmer *et al.*, 2017 ▸; Delgado-Jaime *et al.*, 2013 ▸), geology (Weis *et al.*, 2019 ▸; Atkins *et al.*, 2015 ▸, 2012 ▸), catalysis (Singh *et al.*, 2010 ▸), magnetic materials (Vankó *et al.*, 2007 ▸) and many more (Bauer, 2014 ▸). In VtC-XES, the 1*s* electron is non-resonantly excited into the continuum, and the radiative HOMO (highest-occupied molecular orbitals) to 1*s* channel is detected. Therefore, VtC-XES is highly sensitive to the ligands coordinating to a metal center in complexes or the coordinating ions in solid-state materials. In contrast, in CtC-XES, the relaxation of a core electron from the 2*p* or 3*p* level is recorded, which yields a detailed view on the localized *d*-electron density due to the exchange interaction with the *d* states (Glatzel & Bergmann, 2005 ▸). As such, both methods are capable of removing certain limitations of classical X-ray absorption spectroscopy concerning the discrimination of coordinating atoms that are close neighbors in the periodic table and the determination of oxidation states.

Moreover, XES is the technical basis for resonant X-ray emission spectroscopy studies (RXES) (Glatzel *et al.*, 2009 ▸; Szlachetko *et al.*, 2013 ▸) and high-energy resolution fluorescence detected XAS (HERFD-XAS) (Atkins *et al.*, 2012 ▸, 2015 ▸), the latter demanding an experimental resolution that is lower than the natural lifetime broadening of the excited state. Typically, CtC transitions are used for both resonant inelastic X-ray scattering (RIXS) and HERFD-XAS due to the high transition probability, which is beneficial for recording weak RIXS signals.

For simultaneous X-ray emission-line measurement with high energy resolution, the dispersion of X-rays by a crystal or crystal array is mandatory. For this purpose, wavelength-dispersive spectrometers are used. They typically employ a curved crystal geometry (Johann, 1931 ▸; von Hámos, 1932 ▸). Since bending, on the one hand, improves the photon efficiency but, on the other, reduces the energy resolution, a relatively large bending radius has to be used. It is then possible to achieve a spectral resolution better than 1 eV (Hämäläinen *et al.*, 1995 ▸; Kleymenov *et al.*, 2011 ▸). Such a resolution opens up new perspectives for investigations of chemical reactions. As an example, hydrogen ligands in photochemical water splitting or other catalytic transformations can be investigated under reaction conditions, which is impossible with most other techniques (Burkhardt *et al.*, 2017 ▸). Aside from such specialized examples, in particular, chemical reactions of sustainable character benefit to a large extent from high-resolution X-ray spectroscopy (Zimmer *et al.*, 2017 ▸, 2018 ▸). This aspect becomes even more relevant because of recent developments at X-ray free-electron lasers (Kwan *et al.*, 1977 ▸). With the outstanding time resolution at such facilities, the elementary steps of photoinduced reactions, such as transformations driven by solar light, can be resolved and the geometric plus electronic changes become available (Haldrup *et al.*, 2016 ▸; Alonso-Mori *et al.*, 2012 ▸).

We report on a wavelength-dispersive von Hamos-type spectrometer based on up to 16 crystals and two two-dimensional detector modules installed on beamline P64 at PETRA III. The spectrometer setup allows the emission energy range 50–300 eV to be recorded; its performance is demonstrated by selected examples related to sustainable chemical processes.

## 2. Technical description   

The spectrometer consists of three components: sample area, analyzer crystal array and detector positioning system. The sample area has a sufficiently large space and can accept various sample environments, *e.g.* LHe cryostat, oven, chemical reactors, *etc*. The only limitations are the sample-to-detector distance in the beam direction, and that the output window needs to be sufficiently large to match the solid angle covered by the analyzer crystals.

**Figure 1:**
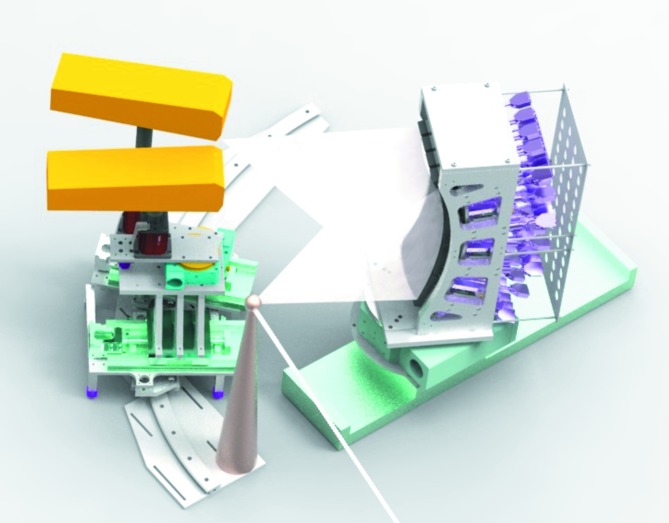
Three-dimensional mechanical drawing of the von Hamos spectrometer equipped with two detectors.

The analyzer crystal holder is placed in a 90° configuration to the incident beam direction along the horizontal plane (Fig. 1 ▸). Such a configuration reduces elastic scattering. The analyzer crystal holder is fixed on a linear translation and a rotation stage for an easy change between spectrometer configurations, covering the angular range ∼85–50°. The analyzer crystal holder is designed for 16 crystal holders in a 4 × 4 array; each crystal holder is equipped with three actuators to allow linear and tilting motions. In the vertical plane, the analyzer crystals are positioned on a cylinder surface with a curvature radius of 500 mm. In the horizontal plane the crystals are linearly displaced. The analyzer crystal holder was designed for analyzer crystals with dimensions of up to 100 mm × 40 mm, and they are cylindrically bent to a radius of 500 mm. Because of its modular design, the spectrometer can also be potentially used with analyzer crystals with smaller or larger radii of curvature.

**Figure 2:**
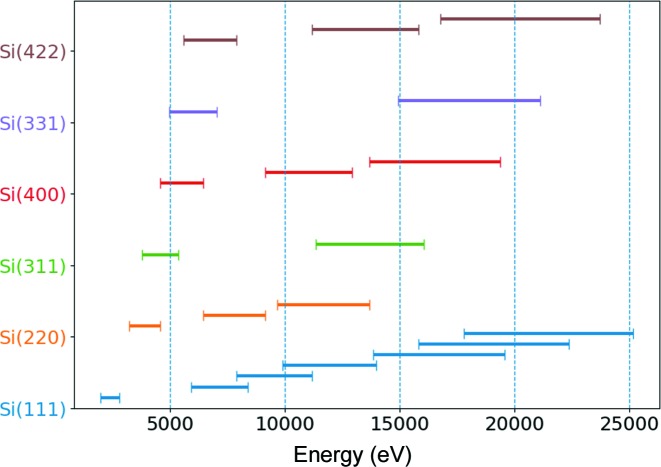
Energy ranges for the available crystal collection [Si(111), Si(220), Si(311), Si(400), Si(331), Si(442)] accounting for their various high-order allowed reflections. These energies can be analyzed within the 85° and 50° angular range of the spectrometer.

For capturing fluorescence lines within the 5–20 keV energy range, an extended collection of crystal analyzers is available. Up to now, the available crystal cuts are Si(111), Si(220), Si(311), Si(400), Si(331) and Si(422). In Fig. 2 ▸, the energy ranges captured with the available crystals (accounting for their various allowed high-order reflections) are displayed with horizontal bars. The energy range covers *K* emission lines from Ti to Ru and *L* emission lines from Ce to Bi. The Rowland circle is set on the vertical plane, thus the beam on the detector is focused in the vertical plane and X-rays are dispersed in the horizontal plane.

The signals from all analyzer crystals can be recorded by one or two detectors simultaneously. The detectors are located on a specially designed detector positioning system. The basis of that system is a curved rail, which allows precise detector positioning at the desired place, corresponding to the chosen Bragg angle. Both detector holders are equipped with motorized linear and rotation stages, and a stand with a platform, allowing additional manual vertical and horizontal displacements. Available displacements allow flexible positioning of the detector module relative to each other, fine-tuning of the detector chip position, and they assure that the detector-chip plane is parallel to the analyzer crystal plane. The detector positioning system allows the spectrometer to capture Bragg angles between 85° and 50°. The upper limit of Bragg angles depends on the sample environment in use.

The spectrometer is designed to be used with a two-dimensional detector with a sensor width of at least 80 mm. So far, two detectors, XSpectrum Lambda 750K (Pennicard *et al.*, 2012 ▸) and Dectris Pilatus 100K, were used. The main difference between them from a spectrometer perspective is the pixel size, which is one of the components determining the energy resolution. The detector choice might also influence the overall spectrometer efficiency. As a reference the following estimations can be made. For concentrated samples, a typical measurement time for *K*α/*K*β emission is in the seconds range. VtC can take up to 1 h. The typical time for an RXES plane is 10–60 min. However, each case should be discussed individually, due to geometric efficiency at different Bragg angles and analyzer crystal reflections strength. When diluted samples are used, the measurement time is proportional to the concentration. Note that all further presented examples were measured with the XSpectrum Lambda detector.

The spectrometer sample area is equipped with an XYZ-theta-X motorized stages stack, which can be used for positioning of relatively small sample environments. Until now, the spectrometer has been successfully used with a flow liquid cell and liquid jet setups, Linkam cooling/heating stage and electrochemical cell. The main criteria of any sample environment is the horizontal sample-to-detector distance, which depends on the Bragg angle applied.

Since the spectrometer operates at atmospheric pressure, a polypropylene bag filled with helium is employed to increase the signal at the detector. It minimizes intensity attenuation and diffuse scattering of the X-rays as they propagate from the sample to the analyzer crystals and then towards the detector.

The X-ray spectrometer is stationed at the undulator-based P64 beamline of the PETRA III storage ring. Beamline P64 is equipped with a liquid-nitrogen-cooled double-crystal monochromator with two pairs of Si crystals, Si(111) and Si(311). Horizontal and vertical focusing Si mirrors with two areas (uncoated and Rh-coated) are positioned behind the monochromator. The beamline delivers monochromatic X-rays over the energy range 4–45 keV. Under the current PETRA III storage ring operational conditions (electron energy of 6 GeV, current 100 mA in top-off mode) and through the Si(311) [Si(111)] monochromator, an incident beam flux of 2 × 10^12^ photons s^−1^ [1 × 10^13^ photons s^−1^] at 8 keV with an energy resolution of about 300 meV [1100 meV] and a beam size of about 70 µm × 200 µm (V × H) is delivered at the sample position (∼87 m from the source). Depending on the particular requirements, higher incident photon flux with worse resolution or lower flux with better resolution can be used. The beamline is described in detail by Caliebe *et al.* (2019 ▸).

## 3. X-ray tracing and resolution tests   

**Figure 3:**
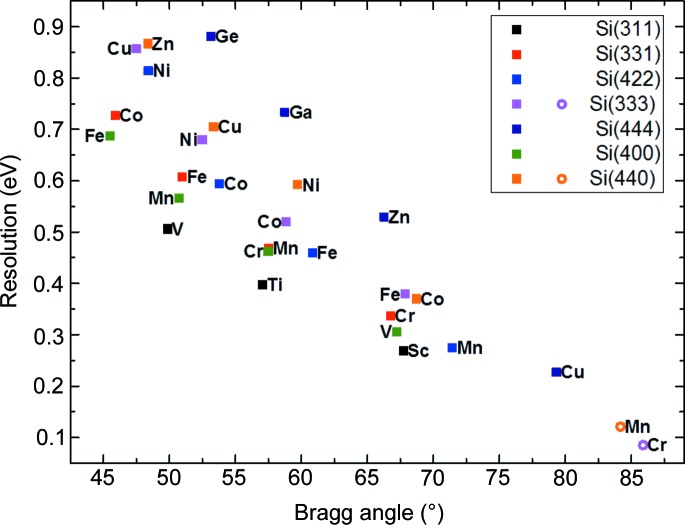
Estimated resolution of the spectrometer at selected *K*α_1_ (solid squares) and *K*β_1_ (open circles) emission lines.

The *XRT* ray-tracing code (Klementiev & Chernikov, 2014 ▸) was used to evaluate the performance of various dispersive geometries. This code is also used to calculate the contributions to the energy broadening, and the energy and intensity distributions when the spectrometer is set to different modes of operation. The theoretical spectrometer resolution was estimated at selected 3*d* element emission lines (see Fig. 3 ▸).

**Figure 4:**
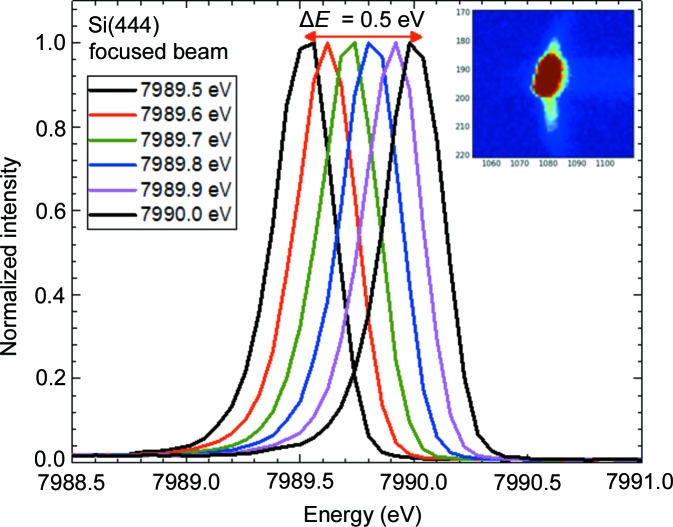
Six elastic scattering scans at 7989.5–7990.0 eV (Bragg angle ≃ 81.8°). The inset shows a picture on the detector at a particular energy, which was used to reconstruct the elastic line.

Spectrometer resolution tests were carried out using the elastic line produced by a focused [70 µm × 200 µm (V × H)] incident beam scattered from Scotch tape. The incident energy was selected using the Si(311) monochromator crystal pair with an intrinsic energy resolution of ∼0.3 eV. The incident energy was varied from 7989 eV to 7990 eV with a step width of 0.1 eV. The spectrometer was set at a scattering angle of 15° to maximize elastic-line intensity. The Si(444) reflection of Si(111) crystals, corresponding to a Bragg angle of ∼81.8°, was used. The inset of Fig. 4 ▸ shows the raw image of the elastic line at selected energies. It should be noted here that it is essential to monochromatize the incident beam with the Si(311) monochromator crystals due to the reduced resolution of the Si(111) monochromator by a factor of three, leading to a horizontal broadening of the beam at the detector. In Fig. 4 ▸ the elastic lines at six different excitation energies are shown; the energy shift is 0.1 eV. Using this result, the conclusion can be drawn that with the presented spectrometer configuration it is possible to detect a fluorescence line shift even if it is ∼0.1 eV. To estimate the experimental resolution we have summed the emission lines with an energy separation of 0.3 eV and 0.4 eV. According to the Rayleigh criterion, two equal-intensity spectral lines can be resolved if the maximum of one line is located at the first minimum of the second line. When the Rayleigh criterion is fulfilled, the peak-to-valley ratio between two lines is ∼0.8. In the case of lines at a 0.3 eV distance, this parameter is ∼0.92, whereas at 0.4 eV it is ∼0.63. That means if we use the resolution criteria of 0.8 peak-to-valley ratio, the obtained experimental resolution will be around 0.34 eV. The obtained number is close to the estimated resolution of the monochromator. Therefore, if an Si(444) analyzer reflection at 8000 eV is used, the resolution is limited by the monochromator only.

## 4. Examples   

The primary scope of the spectrometer is high-energy-resolution X-ray emission measurements. The following examples will show the performance of the spectrometer in various measurement types, *i.e.* CtC and VtC. Taking advantage of the large energy range covered by the analyzer crystals and the multi-crystal design, an example of a two-color experiment is presented; this allows comprehensive studies on samples to be performed without needing to reconfigure the spectrometer. Another example shows a HERFD-XANES reconstruction through measurement of an RXES plane.

### 4.1. X-ray emission measurements   

**Figure 5:**
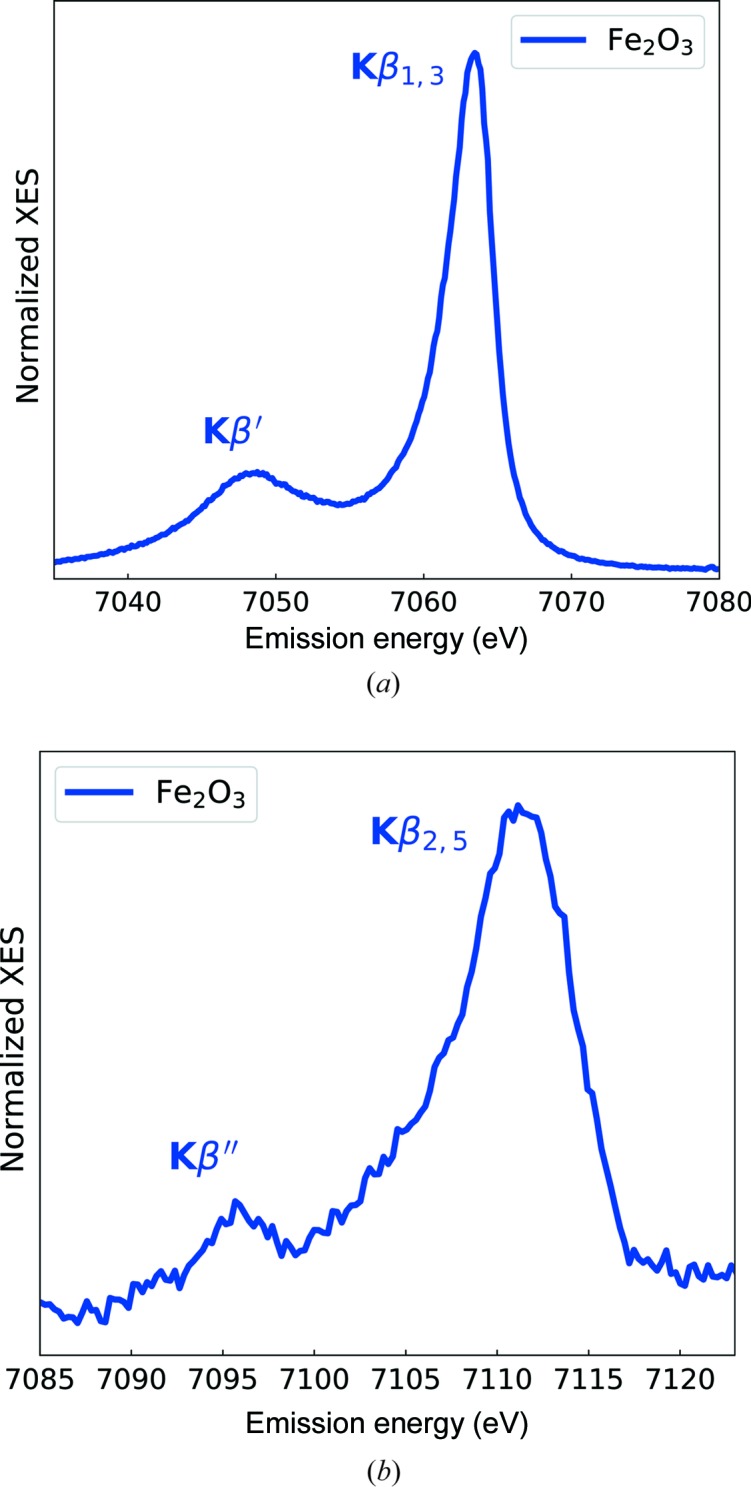
Fe *K*β_1,3_ (top) and Fe *K*β_2,5_ (bottom) spectra of Fe_2_O_3_.

Eight Si(440) crystal analyzers were used to record the Fe *K*β_1,3_ and *K*β_2,5_ XES from Fe_2_O_3_ (Fig. 5 ▸). The spectrometer was set to 66.2° configuration to allow measurement of both Fe *K*β_1,3_ and *K*β_2,5_ emission simultaneously. A cellulose pellet with 1 mg Fe_2_O_3_ powder was prepared in such a way that a 0.3 absorption jump in transmission XAS experiment was achieved, thus self-absorption in fluorescence measurements is negligible. The energy resolution of the experimental setup, measured by the width of the elastic line, was 1.5 eV, including contributions from the Si(311) monochromator and thickness of the pellet. Note that the *K*β_2,5_ emission intensity is 100 times weaker than the *K*β_1,3_ emission.

Due to the multi-crystal spectrometer design, it is possible to perform a so-called two-color experiment (Martinie *et al.*, 2018 ▸) using different analyzer crystals. Each analyzer crystal can be tuned to illuminate distinct areas on the detector, thus separating emission lines from a single chemical element or multiples elements, if a multicomponent compound is studied. If Bragg conditions are rather different and cannot fit the single detector unit, two detector units can be used.

**Figure 6:**
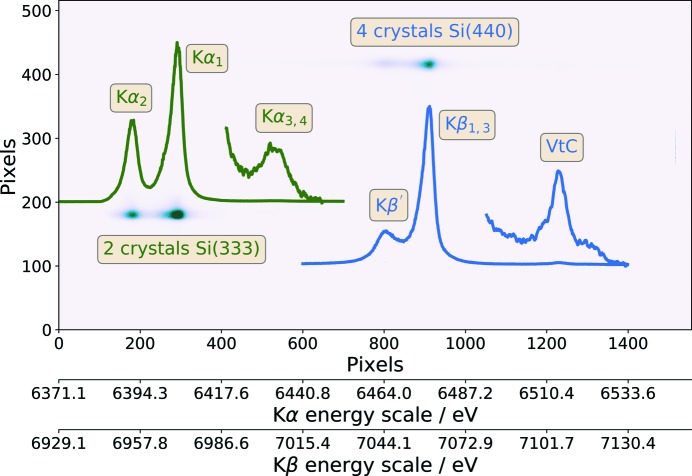
Example of a two-color experiment by measurement of *K*α and *K*β emission lines of Fe(BF_4_)_2_·6H_2_O using a 9300 eV excitation energy. Note that darker spots on the image correspond to higher intensity. Extracted emission signals are plotted on the image. Both *K*α_3,4_ and VtC signals were multiplied by a constant to match the scale of the CtC emission signals. Pixel positions were transformed to energy scales covered by Si(333) and Si(440) reflections.

An example of such a two-color experiment for Fe(BF_4_)_2_·6H_2_O is shown in Fig. 6 ▸. There are two illuminated areas corresponding to Fe *K*α_1,2_ and *K*β, including *K*β′ signals excited by 9300 eV photons. Since the *K*α and *K*β emission lines are separated by more than 600 eV, two types of analyzer crystals [Si(333) and Si(440)] were used to measure these lines simultaneously. However, in this case, it was possible to use a single detector unit because the Bragg conditions for both emission lines were similar. In fact, for the data shown in Fig. 6 ▸, four crystals were focused on the *K*β region and two crystals on the *K*α lines. The low-intensity signal at the higher-energy side of the *K*β emission corresponds to the VtC emission. Additionally, the *K*α_3,4_ satellite signal could be also observed at the high-energy side of the *K*α_1_ emission line.

### 4.2. Resonant X-ray emission and HERFD-XANES   

**Figure 7:**
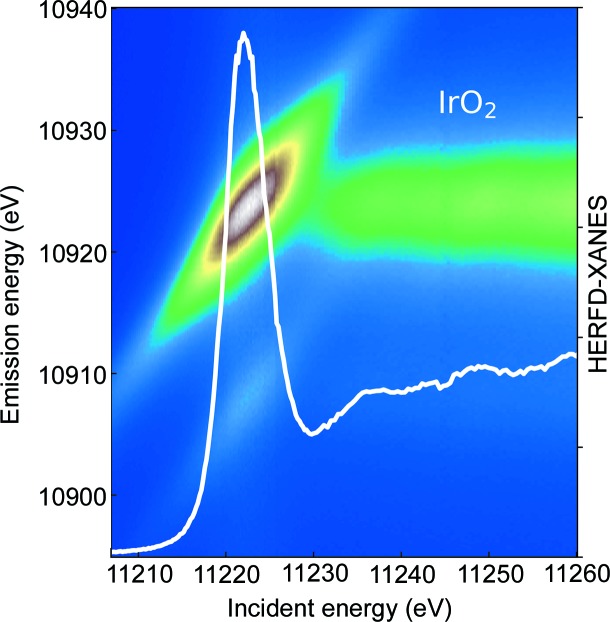
Ir *L*_3_-edge RXES plane and HERFD-XANES spectrum constructed from the plane by a cut at the maximum of the *L*α_1_ emission line.

By variation of the incident energy around the *K*-edge for 3*d* elements or *L*_1,2,3_-edges for 5*d* elements, an RXES plane can be measured. Using appropriate cuts through the RXES plane at particular emission energies, HERFD-XANES can be obtained (Glatzel *et al.*, 2009 ▸). As an example for the data that are obtained with the presented spectrometer, the RXES plane and extracted HERFD-XANES spectrum of IrO_2_ are shown in Fig. 7 ▸. The Ir *L*β_2_ emission line was recorded using a set of eight Si(660) analyzer crystals at a 62.5° Bragg angle. IrO_2_ powder was mixed with ethanol and put on a thin microscope slide to achieve a thin oxide layer after ethanol evaporation. The total measurement time of the RXES plane was 60 min, with a 15 s acquisition time for a single emission measurement. The energy resolution, determined from the full width at half-maximum of the elastic line, was ∼1.78 eV.

## 5. Conclusions   

A wavelength-dispersive von Hamos-type spectrometer has been installed at beamline P64 of the PETRA III synchrotron radiation facility and highly benefits from the high photon flux produced by the undulator source at the beamline. The spectrometer is designed to employ up to 16 analyzer crystals and two position-sensitive detector modules. A combination of available crystal sets and use of two detectors allow the simultaneous acquisition of at least two emission lines. An experimental resolution of 0.35 eV was measured at 80° configuration using Si(444) analyzer crystals. The combination of multi analyzer crystal design, high resolution and high photon flux at beamline P64 allows measurement of even very weak signals.
